# Tissue specific expression of UMAMIT amino acid transporters in wheat

**DOI:** 10.1038/s41598-021-04284-7

**Published:** 2022-01-10

**Authors:** Ze Tian Fang, Rajan Kapoor, Aniruddha Datta, Sakiko Okumoto

**Affiliations:** 1 Department of Soil and Crop Sciences, Texas A&M University and Texas A&M AgriLife Research, College Station, TX USA; 2 Department of Electrical and Computer Engineering, Texas A&M University, College Station, TX 77843 USA

**Keywords:** Plant molecular biology, Plant physiology

## Abstract

Wheat grain protein content and composition are important for its end-use quality. Protein synthesis during the grain filling phase is supported by the amino acids remobilized from the vegetative tissue, the process in which both amino acid importers and exporters are expected to be involved. Previous studies identified amino acid importers that might function in the amino acid remobilization in wheat. However, the amino acid exporters involved in this process have been unexplored so far. In this study, we have curated the Usually Multiple Amino acids Move In and out Transporter (UMAMIT) family of transporters in wheat. As expected, the majority of UMAMITs were found as triads in the A, B, and D genomes of wheat. Expression analysis using publicly available data sets identified groups of TaUMAMITs expressed in root, leaf, spike, stem and grain tissues, many of which were temporarily regulated. Strong expression of TaUMAMITs was detected in the late senescing leaves and transfer cells in grains, both of which are the expected site of apoplastic amino acid transport during grain filling. Biochemical characterization of selected TaUMAMITs revealed that TaUMAMIT17 shows a strong amino acid export activity and might play a role in amino acid transfer to the grains.

## Introduction

Amino acids serve as the major carrier of nitrogen within the long-distance transport systems (i.e. xylem and phloem) of plants. Amino acids, typically synthesized in the photosynthetic leaf tissues from inorganic N, are carried to heterotrophic tissues such as seeds and roots. Such exchanges of amino acids between organs are indispensable for the plant growth.

Due to their zwitterionic nature, the passing of amino acids through cellular membranes is mediated by dedicated carrier proteins. In the past three decades, many of the amino acid transporters involved in such processes have been identified. Cellular amino acid importers belonging to the families of Amino Acid Permeases (AAPs), Lysine and Histidine Transporters (LHTs), Cationic Amino acid Transporters (CATs), Proline and GABA transporters (ProT/GABAT), Auxin Transporters (AUXs), and Aromatic and Neutral amino acid transporters (ANTs) have been identified, and for some transporters, their physiological roles have been identified, including amino acid export from source leaves^1^, amino acid loading to the seeds^2–4^, xylem-to-phloem amino acid transfer^5^, amino acid transport from the nodules^6^, and root uptake of amino acids^1,7–10^. These transporters belong to amino acid -polyamine-choline (APC) family and the amino acid/auxin permease (AAAP) family, and most of them function as amino acid-proton symporters^11,12^. Another and more recently identified family, UMAMITs, encode bidirectional amino acid transporters and vacuolar auxin transporters. Although this family of transporters has not been studied as extensively as the members of APC and AAAP superfamilies, the physiological roles of some of the UMAMIT transporters have been identified. In Arabidopsis, UMAMIT11, 14, 18, 24, 25, 28 and 29 have been implicated in seed loading^13,14^, UMAMIT14 and 18 in phloem export and root amino acid secretion^15,16^, and WAT1/UMAMIT5 is involved in vacuolar transport of auxin^17^. The major functional difference between APC/AAAP family transporters and UMAMIT is that UMAMIT is capable of functioning as cellular amino acid exporters^13,15^.

Using bioinformatics and the ever-increasing number of available plant genomes, it has been revealed that the APC, AAAP, and UMAMIT families of transporters are conserved among all higher plants investigated so far, the vast majority of which are uncharacterized. An inventory of these amino acid transporters in the crop of interest can be used as the starting point to modify and optimize the amino acid transport within the plant, a strategy with a record of success in multiple model and crop species^3,5,18^.

Among cereals, amino acid transport in wheat is of specific interest due to the wheat flour’s end-use. The dough quality is tightly linked to the quantity and quality of its storage proteins in the endosperm, glutenins and gliadins, which determines the viscosity and elasticity^1–3^. Accumulation of glutenins and gliadins relies on amino acids supplied during the seed filling phase, and increased N supply during the seed filling stage is associated with higher grain protein content. Therefore, amino acid transporters expressed during this stage are of particular interest. Previous publications have already identified members of the APC and AAAP superfamilies in wheat, and identified the transporters that could be involved in seed loading^19,20^. Here we report the identification of potential amino acid transporters belonging to the UMAMIT family in wheat. We have identified 213 putative members, and ~ 70% of the members were found as triads on all three (A, B, and D) genomes. Further, spatio-temporal analysis of UMAMIT expression revealed that many are developmentally controlled, and are expressed during the seed filling stage in either developing kernels or source leaves, suggesting that the function of UMAMITs in seed filling might be conserved in wheat.

## Results and discussion

### Identification of TaUMAMIT genes

Wheat UMAMIT homologs were searched using Interpro and Pfam programs^21,22^. Interpro search of all 44 Arabidopsis UMAMIT genes classified them as WAT1-related family (IPR30183), and most carried EamA domain (IPR00620). Searching wheat proteins (Ensembl v47) for IPR30183 and IPR00620 yielded 194 and 192 translated sequences respectively. Among those, 182 sequences belonged to WAT1-related family (IPR30183) and contained EamA domain (IPR00620). Pfam search of Arabidopsis UMAMIT genes identified two repeats of PF00892 motifs in full-length proteins (EamA domain). Pfam search of wheat proteins with PF00892 yielded an identical list of 194 proteins with the Interpro search using IPR00620. Blastp search using all AtUMAMIT genes, at the e-value cutoff of 1e−5, yielded 186 entries, all of which were included in the list found in the Interpro search. In total, 204 genes have been identified. The domain structure of full-length UMAMITs is shown in Fig. S1.

In addition, low confidence genes in the Refseq v1.1 cDNA annotation have also been mined. A tblastn search using all AtUMAMIT protein sequences at e-value cutoff of 1e−5, yielded an additional 28 genes, of which 12 were partial genes. Corresponding intervals for all low confidence proteins were identified in IWGSCv1.0 genome, and gene models were examined using the reads mapped to the corresponding intervals in CRAM files for 308 publicly available RNAseq datasets (Table S1). Of the 28 low confidence genes, 10 were significantly expressed, allowing the gene models to be examined. The longest isoform for each was chosen and searched for conserved domains with Interpro. Nine out of 10 included domain IPR30184 (WAT1-related). These proteins were added to the list of high confidence proteins, resulting in the list of 213 TaUMAMIT genes. As expected for hexaploidy wheat, the majority of genes were found in all three subgenomes; among 213 proteins, 153 were found as triads, whereas 60 were not found in at least one of the three genomes (Table S2). In total, 98 distinct groups of homeologs were identified, among which 51 were found in triads (Table S3).

**Figure 1:**
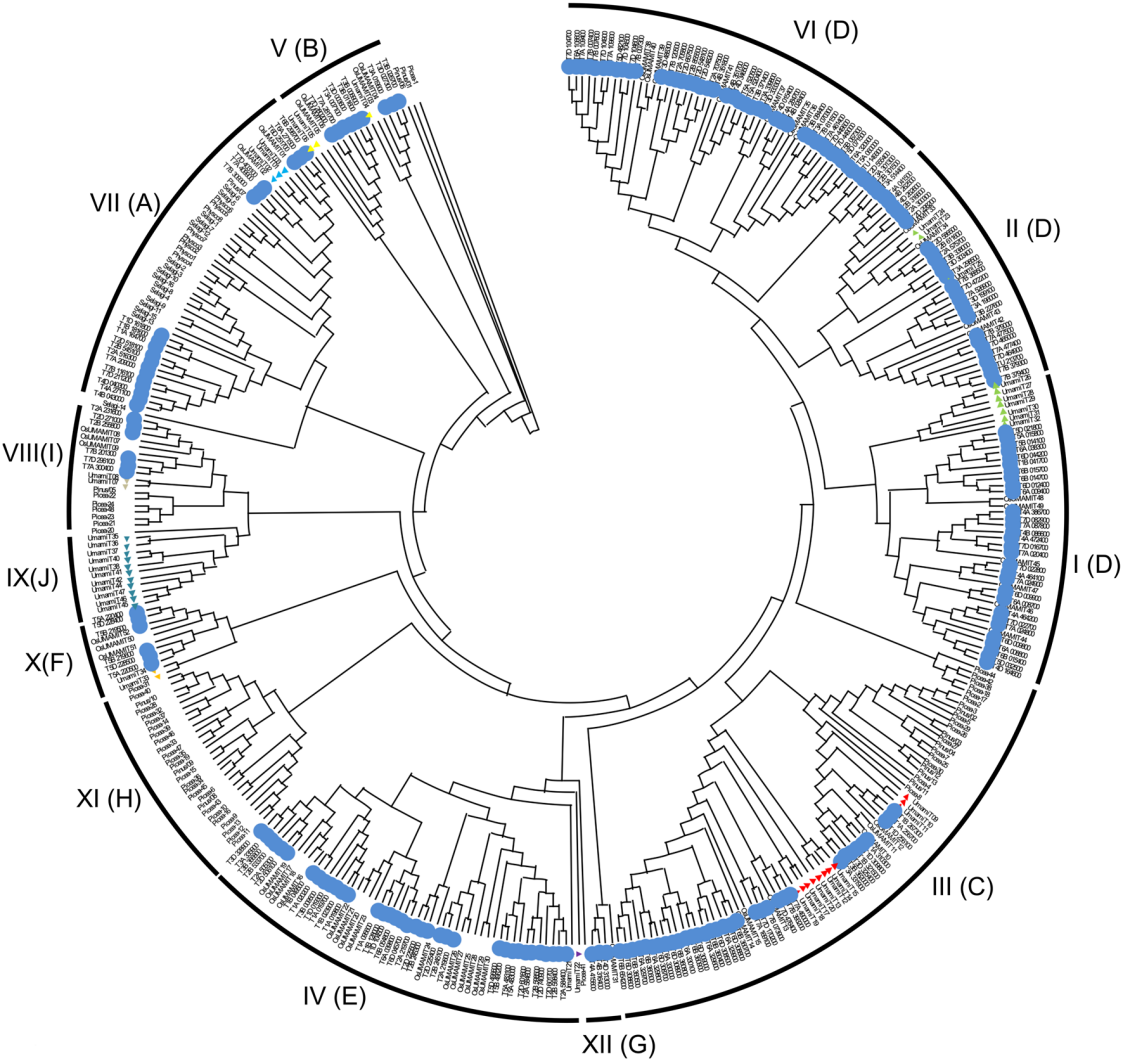
Phylogneitc relationship between UMAMITs of wheat and other species. The wheat gene IDs are abbreviated as; TraesCS1A02G019700 = Ta1A019700, indicated by blue dots. UMAMITs from other species are indicated as follows: Arabidopsis thaliana, no abbreviations (UMAMIT); Physco, *Physcomitrella patens*; Selagi, *Selaginella moellendorffii*; Pinus, *Pinus pinaster*; Picea, *Picea abies*; Os, *Oryza sativa*. Arabidopsis UMAMITs are indicated with triangles. The clade IDs have been assigned according to the numbers based previous publications^13,24^. The corresponding clade in Zhao et al. is indicated in parenthesis^25^.

**Figure 2:**
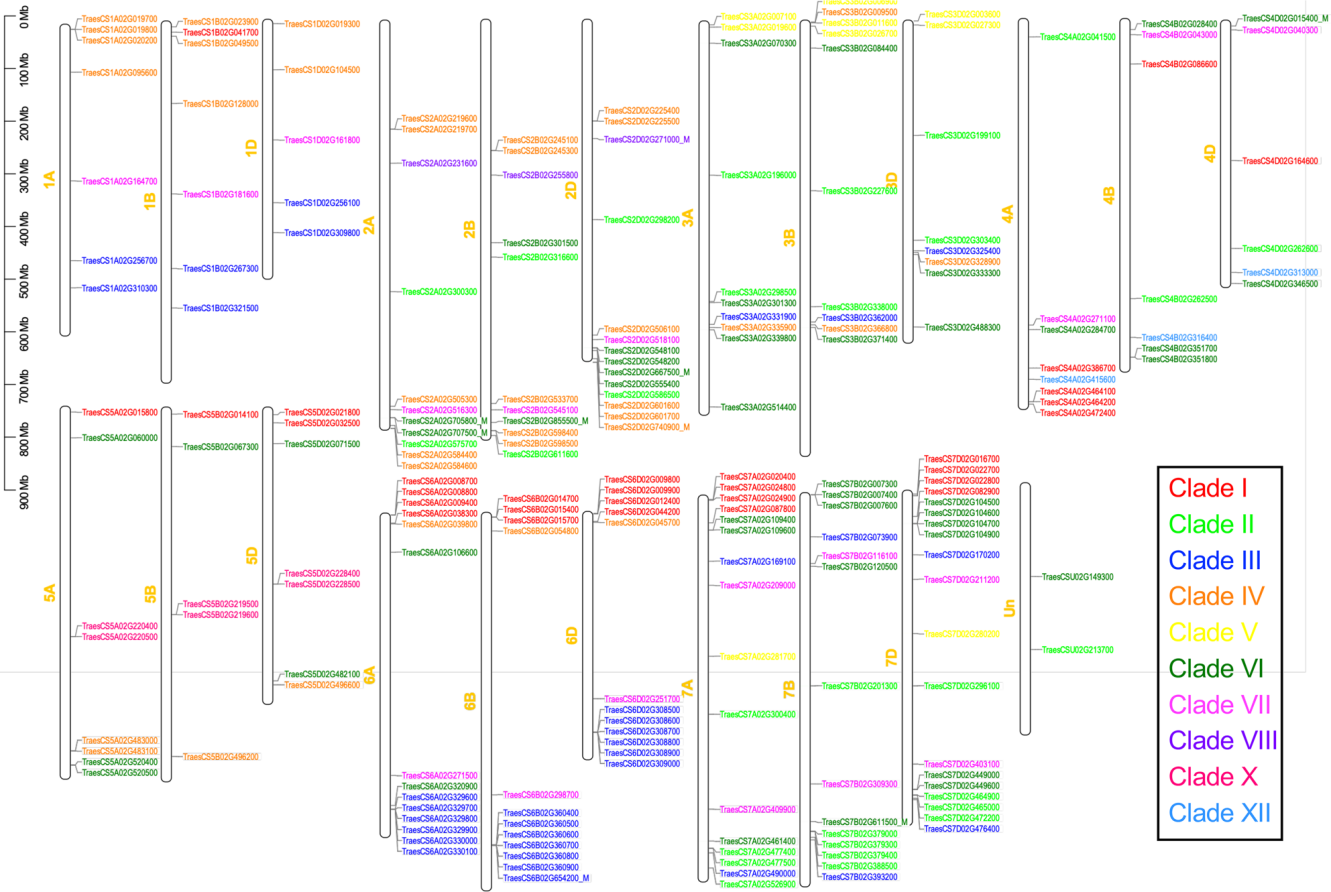
Chromosomal locations of TaUMAMIT genes. The gene IDs are color coded by the clades as indicated in the inset. Low confidence genes identified in this study are indicated with _M. Un represents the unassigned scaffold.

Most of the UMAMIT family proteins analyzed so far are broad specificity amino acid transporters, except for Arabidopsis WAT1, which is a vacuolar auxin transporter^17,23^. To identify the clades to which the TaUMAMIT proteins belong, a phylogenetic tree including the sequences from six representative species (sequences found in Table S4) was constructed (Fig. 1). Previously published phylogenetic analyses of AtUMAMITs identified seven clades^13,24^, and a more recent study identified three additional clades while merging the previously identified clades I, II, and VI into a single clade (D)^25^. Genes belonging to clades I to IV, VII, IX, and X were found in both Arabidopsis and wheat. Clades I and III includes previously characterized amino acid transporters^13–16^. Two other clades include previously documented genes; clade V includes Arabidopsis WAT1^17,23^, and clade IX includes RTP1/UMMAIT36, which has been identified as a negative regulator of SA-mediated pathogen response^26^. Clade VI, which contains many wheat and rice UMAMIT members, did not contain any Arabidopsis members. Clade IX, on the other hand, did not contain any UMAMIT from wheat, confirming the previous report showing that this clade is dicot-specific. In summary, all but one clade of UMAMITs found in Arabidopsis were conserved in wheat. TaUMAMIT genes were distributed on all 21 chromosomes and the unassigned scaffold. Some of the TaUMAMITs were found in multiple tandem duplications, such as the lower arm of chromosome 6, where 6 or 7 tandem repeats of TaUMAMITs belonging to clade III are found (Fig. 2). In some cases, subgenome-specific duplication was also observed. Overall, the results suggest that UMAMITs have been mostly conserved since the formation of hexaploidy in wheat.

According to the positions in the phylogenetic trees, we have assigned names to the gene models (Tables S2 and S3).

### Tissue expression of UMAMITs

**Figure 3:**
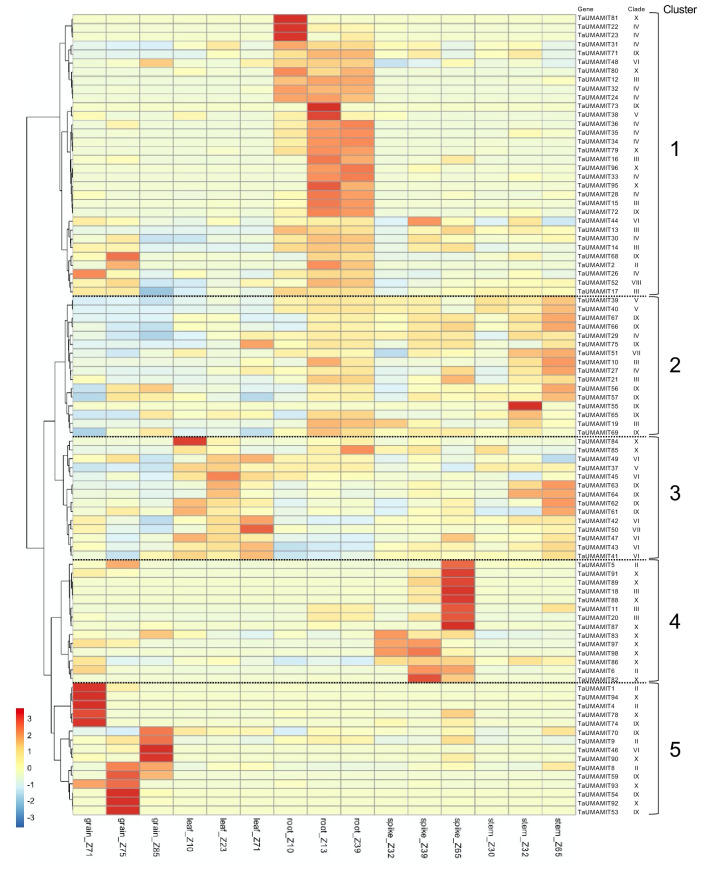
Expression of UMAMIT genes in different organs. The heatmap is scaled by row (each gene). For each gene, expression values were averaged for all representing wheat subgenomes. Growth stages are expressed in Zadoks growth scale (i.e. Z71 represents Zadoks stage 71). UMAMIT genes without expression in any of the cell type has been excluded from the figure.

In order to understand the tissue specificity of TaUMAMITs, an RNAseq dataset prepared from grain, leaf, root, spike and stem tissues at three different developmental stages each^4^ was aligned to the IWGSCv1.0 genome. Reads mapped to the UMAMIT genes annotated in the published transcriptome (v. 47), as well as to the manually annotated UMAMITs identified in this study were counted. In total, the expression of 91 out of 98 homeologous groups was detected in at least one tissue. The largest cluster of genes (cluster 1 in Fig. 3) was mainly expressed in the roots. Some genes such as TaUMAMIT22 and 23 were specific to an early stage of development (Z10, first leaf through coleoptile), whereas the majority of genes in this group showed higher expression at later stages (Z13, three leaves stage; Z39, flag leaf stage) of development. The homeologous groups expressed the most were TaUMAMIT17 and 39 (average TPM through three stages at 968.4 and 676.9, respectively), which were expressed highly in all three developmental stages. Homeologous groups belonging to cluster 2 are largely specific to stem tissues, most of which were expressed higher in the later stage (Z65, during anthesis) compared to the earlier stage (Z32, second node detectable). Likewise, many genes that are mainly expressed in leaves (cluster 3) are induced during the grain filling (Z71), as observed for TaUMAMIT50 and TaUMAMIT42. Together, these TaUMAMITs induced during reproductive growth in the vegetative tissues could be involved in nitrogen remobilization. Clusters 4 and 5 genes are expressed mainly in the reproductive tissues (spikes and grains, respectively). Multiple homeologous groups belonging to clade III (Figs. 1, 3, TaUMAMIT11, 18, 20 and 21) are highly specific to the spikes at Z65 (mid anthesis, early grain development). Clade III genes in Arabidopsis have been previously shown to be involved in grain filling^5,6^, therefore the function of clade III UMAMITs might be conserved between Arabidopsis and wheat. Likewise, TaUMAMIT1, 4, 5, 6, 8 and 9, belonging to clade II that includes previously characterized AtUMAMITs involved in grain filling^7^, were found to be expressed at water ripe, medium milk and soft dough stages of grain filling (Z71 Z75,and Z85 respectively).

### Grain development

**Figure 4:**
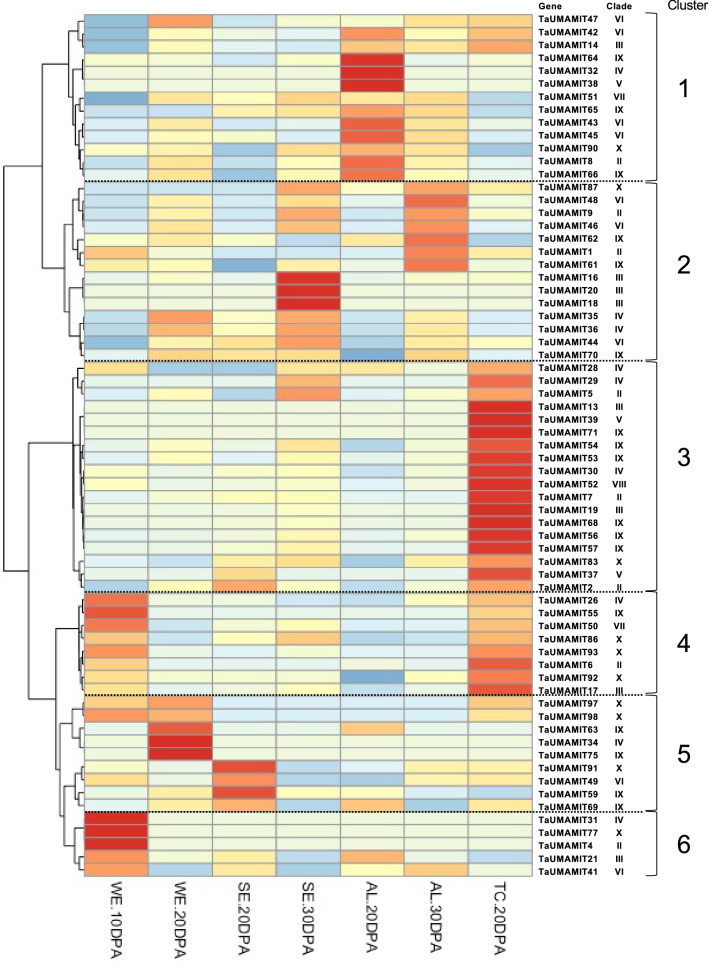
Expression of UMAMIT genes in different cell types of developing grains. The heatmap is scaled by row (each gene). For each gene, the expression values were averaged for all representing wheat subgenomes. *WE* Whole endosperm, *WE* Starchy endosperm, *AL* Aleurone layer, *TC* Transfer cells. UMAMIT genes without expression in any of the cell type has been excluded from the figure.

Amino acids are the main carrier of N delivered to the developing seeds via the phloem^8^. In wheat, amino acids are released from the nucellar projection on the maternal side to be picked up by the filial tissues such as the embryo, transfer cells and the aleurone layer surrounding the endosperm^9^. To identify the UMAMITs involved in the amino acid transfer process described above, a publicly available RNAseq data set derived from whole endosperm at 10 days post-anthesis (DPA), starchy endosperm at 20 and 30 DPA, aleurone layer at 20 and 30 DPA, and transfer cells at 20DPA^10^ were queried as described above. The cell type that expressed the largest number of UMAMIT genes was transfer cells (Fig. 4, cluster 3), with 18 genes being largely specific to this cell type. Cluster 4 genes were also expressed in transfer cells, with expression detected in 10DPA whole endosperm. TaUMAMIT92 and 17 were the most highly expressed triads in the transfer cells (average TPM 264.5 and 96.1, respectively). Another cell type with prominent UMAMIT expression was aleurone layer cells, with 14 genes largely specific to this cell type (Fig. 4 cluster 5). TaUMAMIT51 and 43 were the most abundant triads in aleurone cells (72.9 and 45.9 TPM, respectively in 20 DPA). Starchy endosperm cells also expressed several UMAMITs, most of which were also developmentally regulated (Fig. 4 clusters 2 and 5).

Previous studies found transfer cells and aleurone layer cells to be also expressing multiple amino acid importers, signifying the potential role for these cell types in amino acid transport^11^. The expression of concentrative amino acid importers and UMAMITs within the same tissue type begs a question; since UMAMITs characterized so far function as bidirectional transporters^5–7,12^, the co-expression of UMAMIT transporters and concentrative amino acid importers could potentially lead to a futile cycle in which amino acid entering the cell through an amino acid importer is lost through UMAMIT transporters. However, the currently available data set does not allow for cellular resolution. Likely, the tissues examined consist of more than one cell type with distinct functions, as revealed by recent single-cell RNAseq studies showing that the cells in the vascular bundle expressing mainly sugar and amino acid importers are distinct from those expressing the exporters^13,14^. In addition, polar localization of transporters within the same cell type might allow for the directional transport, as previously found for both plant^15,16,18^ and animal^19,20^ systems. Some of the UMAMITs might also be involved in amino acid transport across the intracellular membranes, analogous to AtUMAMIT24^7^. Additional studies of UMAMIT localization within this tissue type will help to understand the path of amino acid transport and the roles of UMAMIT.

### Senescence

**Figure 5:**
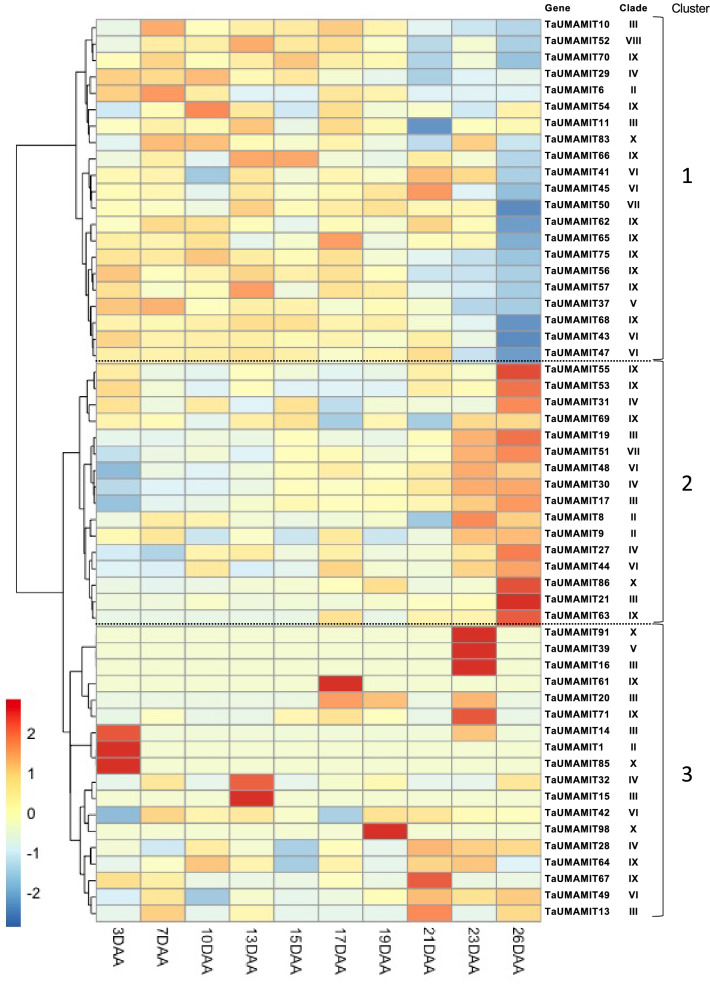
TaUMAMIT expression in senescing leaves. The heatmap is scaled by row (each gene). For each gene, the expression values were averaged for all representing wheat subgenomes. *DAA* Days after anthesis.

During grain filling, as much as 90% of nitrogen is remobilized from the leaves to the developing grains^21^, and remobilized N accounts for ~ 70% of total grain N^22^. During reproductive growth, RuBisCO and other chloroplastic proteins in the source leaves are degraded to amino acids, which are transported to the grains^23^. Therefore, high amino acid export activity is expected at the source leaves during grain filling. To identify the TaUMAMITs potentially involved in amino acid export from senescing leaves, RNAseq data set including post-anthesis leaves was queried^17^. Among 55 homeologous groups expressed, 16 groups (Fig. 5, cluster 2) were induced at the 23 and 26 days after anthesis (DAA), at which stage chlorophyll contents decrease significantly^17^. The second cluster (Fig. 5, cluster 1) on the other hand, showed higher expression in the earlier stages and was downregulated during senescence.

These results, therefore, seem to show that the UMAMIT genes involved in the amino acid export from actively photosynthesizing leaves are likely different from those mediating the export at the later stage of senescence. Those TaUMAMITs upregulated in the late senescence stage might be a part of the gene network mediated by a NAC transcription factors such as NAM-B1, which promotes senescence and increases N transfer to the grains^27,28^, or other pathways induced during cell death^29^. Interestingly, an ectopic expression of Arabidopsis UMAMIT has been shown to induce SA-mediated stress response and cell death^30^. Whether TaUMAMITs are expressed in response to senescence, or involved in promoting cell death in the senescing tissues remains to be seen.

Earlier studies examining the leaf N content after anthesis showed a gradual decline in N throughout the filling stage and not a sharp decline at the late senescing stage^31–33^, and phloem amino acid content remains high (~ 900 mM detected in aphid stylet exudation) throughout early and mid grain filling stages despite some changes in composition^34^. Therefore, it is likely that amino acid export out of the flag leaf is also a continuous process, and is mediated not only by those expressed in the late senescence stage but also by those expressed in the earlier stages. Understanding the main players in leaf N export will require additional biochemical analysis to identify the active amino acid exporters, as well as detailed temporal analysis of leaf N loss and the expression profile of TaUMAMITs.

### Expression and amino acid transport activity of selected TaUMAMITs expressed in grains

**Figure 6:**
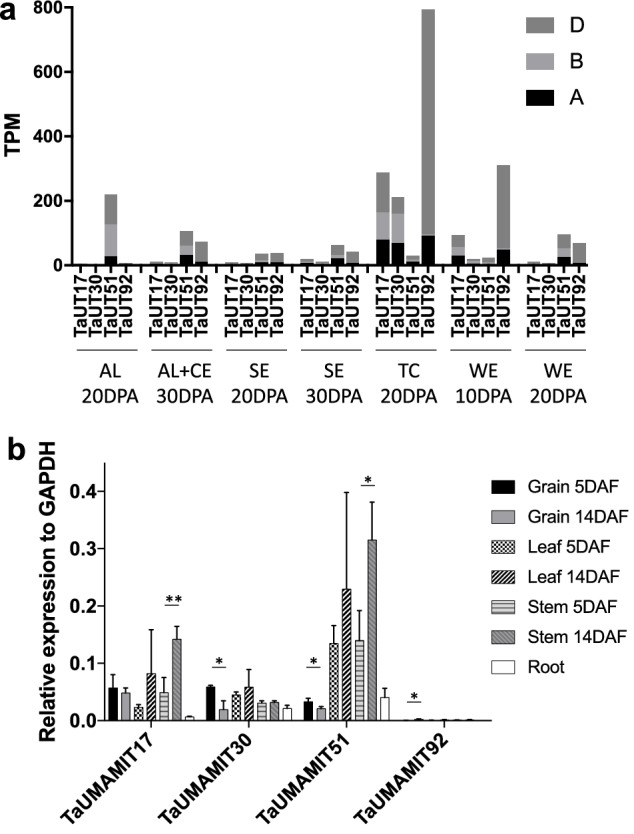
Expression of selected TaUMAMITs in grain tissues. (**a**) Expression of TaUMAMIT17, 30, 51, 92 in various grain tissues. The TPM values for each subgenome are indicated. *AL* Aleurone layer, *CE* Central endosperm, *SE* Starchy endosperm, *TC* Transfer cells, *WE* Whole endosperm, *DPA* Days after anthesis. (**b**) Expression of selected TaUMAMITs in developing grains, leaf, stem and root. The mRNA levels detected with RT-qPCR are expressed as the relative amount to the reference gene, TaGAPDH. Statistical difference according to Student’s t-test is indicated by asterisk (*p* < 0.05). All experiments were conducted at n = 3. *DAF* Days after flowering.

To validate the expression of some of the TaUMAMITs in grains, four strongly expressed TaUMAMIT genes were selected (Fig. 6a). The expression pattern of TaUMAMIT17 (clade III), 30 (clade IV), 51 (clade VII), and 92 (clade VI) was examined using qRT-PCR, using a primer set that detects all three homeologs. As expected, all of them were significantly expressed in grains at both 5 and 14 days after flowering (DAF), representing early- and mid-grain filling stages, respectively (Fig. 6b). TaUMAMIT17 accumulation was similar between five and 14 DAF. For TaUMAMIT30, 51, and 92, the expression levels differed between the two time points; TaUMAMIT30 and 51 expression was higher at 5DAF whereas the level of TaUMAMIT92 was higher at 14DAF (Fig. 6b). In addition, TaUMAMIT17, 30 and 51 were expressed in leaf, stem and root tissues. The expression level of TaUMAMIT17 and 51 in the stem was higher at 14 DAF compared to 5 DAF (Fig. 6b).

**Figure 7:**
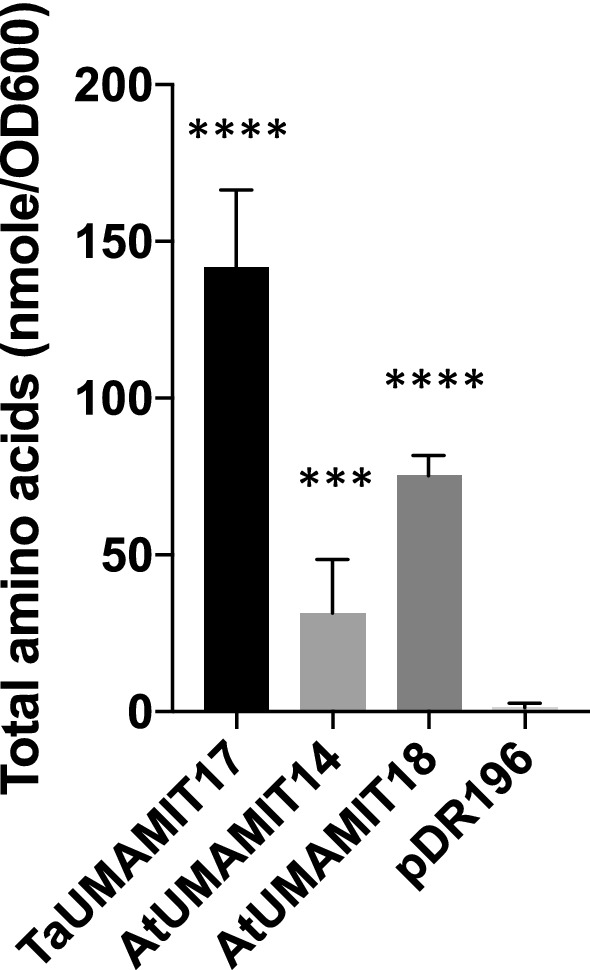
TaUMAMIT17 exports amino acid secretion in yeast. Amino acid secretion from 22Δ10α cells expressing TaUMAMIT17D, AtUMAMIT14 and AtUMAMIT18, compared the vector control (pDR196). Statistical difference according to Student’s t-test is shown with asterisks (n ≥ 5).

To test their functions as amino acid exporters, one homeolog each of TaUMAMIT17, 30, 51, 92 were selected (Table S5) and expressed in the yeast strain 22Δ10α which lacks all known endogenous amino acid importers^12^. The cells were grown in a minimal medium containing ammonium as the sole nitrogen source, and the excretion of L-amino acids to the medium was measured using a colorimetric assay. The results indicated that TaUMAMIT17 has a strong activity for amino acid export, resulting in > 100 fold concentration of amino acids in the growth media compared to the control expressing the empty vector, and higher than cells expressing previously characterized AtUMAMIT14 and 18 genes (Fig. 7). TaUMAMIT17 belongs to clade III, to which many Arabidopsis UMAMITs with known amino acid export activity belong^5,6,12^. Expression of TaUMAMIT30 (clade IV) and 51 (clade VII) did not result in a statistically significant increase in amino acid excretion compared to the WT, despite them belonging to the clades that include Arabidopsis members that show some activity as amino acid exporters^25^.The amino acid secretion from yeast expressing clade I, V, and VII transporters was lower than those expressing clade III genes from Arabidopsis in the yeast expression system. TaUMAMIT92 belongs to clade VI, which largely consists of monocot members. TaUMAMIT92 did not show a significant amino acid transport activity in our assay. Therefore, whether transporters in these clades indeed function as amino acid exporters remain to be seen.

## Methods

### Phylogenetic analysis

The longest isoforms of wheat UMAMIT genes (213 total) and Arabidopsis UMAMIT genes (44 total) were used for the analysis. Additionally, UMAMIT genes in four representative species (*Physcomitrella patens, Selaginella moellendorffii, Arabidopsis thaliana, Oryza sativa*) previously published were included^31^. The sequences were aligned using MUSCLE^35^, with gap open penalty of − 2.9, gap extend 0 and hydrophobicity multiplier 1.2, UPGMA clustering option. The aligned sequence was used to build a phylogenetic tree with RAxML version 8.2^36^, using rapid bootstrap analysis (1000 iterations) and GAMMA model of rate heterogeneity using empirical base frequencies and the LG substitution model.

### Expression analysis of TaUMAMIT genes

Wheat RNAseq projects publicly available on NCBI Sequence Read Archive (SRA) database (https://www.ncbi.nlm.nih.gov/sra)36 were surveyed and datasets with the following SRA study IDs were selected for further processing: ERP004714 (spatiotemporal development), ERP004505 (grain development), and SRP166963 (leaf senescence). For each SRA study, the read counts for high confidence UMAMI genes were obtained from iRAP pipeline (v1.0.1)^37^—mapped data available at European Nucleotide Archive (ENA)^38^ (https://www.ebi.ac.uk/ena/browser/home), obtained using RNASeq-er REST API^39^. The pipeline uses FASTX (v0.0.13) for filtering and adapter trimming, FASTQC (v0.11.7) for quality check, HISAT2 (v2.1.0)^40^ for alignment and featureCounts (v1.6.2)^41^ for quantification. The mapping quality for every dataset was confirmed to be above 75%. For determining counts of low confidence UMAMIT genes, the HISAT2^40^ mapped CRAM alignment and corresponding CRAI index files from the ENA database were used to count the reads within the corresponding intervals identified in this study. The wheat genome assembly and GTF version 41 were downloaded from the Ensembl Plants website (ftp://ftp.ensemblgenomes.org/pub/plants/release-41). SAMTools (v1.9)^42^ was used to extract the reads within gene boundaries for the low confidence UMAMIT genes. FeatureCounts^41^ from Subread package (version 1.6.2) was then used with the GTF file corresponding to the low confidence UMAMIT genes to count the mapped reads. The read counts for the high- and low-confidence genes were processed in featureCounts, then normalized using GeTMM^43^, where gene length was obtained by summing lengths of unique exons for each gene from the GTF files. Mean was taken across replicates and the resulting expression was summed for each gene in the triad. For triads missing one or more genes in the triad, only the mean expression of available genes was added. This was followed by log2 transformation in GeTMM with an offset of 1 to reduce the dynamic range for creating the heatmap. The R-package pheatmap (v1.0.12)^44^ was used to generate the heatmaps by scaling the mean sample expression by row to show peak tissue expression. The row clustering was performed using the ward.D method and Euclidean distance^11^. The genes with zero variance across all samples were removed to resolve errors resulting from clustering in pheatmap.

### Yeast amino acid export assay

TaUMAMIT sequences were synthesized (Twist Bioscience, USA) and cloned into the pTwist_ENTR vector. TaUMAMIT17 and 92 were codon-optimized to avoid GC-rich sequences. The sequences of synthesized fragments are found in Table S5. All TaUMAMITs were introduced into the yeast expression vector pDR196-f1GW^45^ via gateway cloning (Invitrogen, USA).

TaUMAMITs amino acid export activity was determined via yeast cell secretion in liquid media according to the procedure previously described^7,12^. Briefly, 22Δ10α (genotype MATα gap1-1 put4-1 uga4-1 can1::HisG lyp1-alp1::HisG hip1::HisG dip5::HisG gnp1Δ agp1Δ ura3-1) expressing individual UMAMIT proteins were grown in a minimum medium^46^ overnight at 28C. On the next day, the cultures were diluted to OD_600_ = 0.1 in 5 mL of minimal medium, then incubated at 28C for 5.5 h. The cultures were spun down and the supernatant was flitered using a 10 kDa exclusion membrane. Amino acid concentration of the supernatants was determined using L-Amino Acid Quantitation Kit (Sigma-Aldrich, USA) following the manufacturer’s instruction.

### Plant materials and growth

Wheat (*Triticum aestivum* cv. TAM114^47^) plants were grown in 2018 at Texas A&M Research and Extension Center in Bushland, TX (35° 06′ N, 102° 27′ W). Standard agronomic practices were performed according to Texas A&M AgriLife extension recommendations. Spikes at five and 14 days after flowering were harvested and stored in liquid N_2_. The grains were mechanically isolated from the glumes while soaking in liquid N_2_, and stored at − 80 °C until use. Flag leaves and stems were harvested from plants grown at 28 °C with a 12 h light cycle in a greenhouse, at five and 14 days after flowering. Root tissues were collected from 10 day-old seedlings germinated on filter paper moistened with distilled water.

### RT-qPCR analysis

Total RNA was extracted using a modified method described previously^48^, followed by a clean-up using Qiagen RNeasy® Plant Mini Kit column and DNaseI (Qiagen, USA) following the manufacturer’s instruction. Complementary DNA synthesis was performed using iScript™ Advanced cDNA Synthesis Kit (Bio-rad, USA), using 2 μg of total RNA as a template. Quantitative PCR was performed using SsoAdvanced™ Univseral SYBR® Green Supermix (Bio-rad, USA) on the CFX96™ Real-Time System platform, using the primers listed in Table S6. Relative expression to the reference gene was calculated as E_t_^−Cqt^/E_r_^−Cqr^, where E_t_ and E_r_ are the efficiencies of the primer set for the target and the reference genes, respectively, and Cqt and Cqr are the Cq values for the target and the reference, respectively. E_t_ and E_r_ values were calculated using LinRegPCR software^49^.

## Supplementary Information

Supplementary Figure S1.

Supplementary Tables.

## Data Availability

The raw RNAseq data used for this publication are publicly available. The list of accession numbers is found in Table S1. The constructs used for this publication are available on request.
