# Supplementary figures and images for: Tissue specific expression of UMAMIT amino acid transporters in wheat
Source: Sci Rep. 2022 Jan 10;12:348. doi: 10.1038/s41598-021-04284-7 (PMC8748447; doi:10.1038/s41598-021-04284-7)

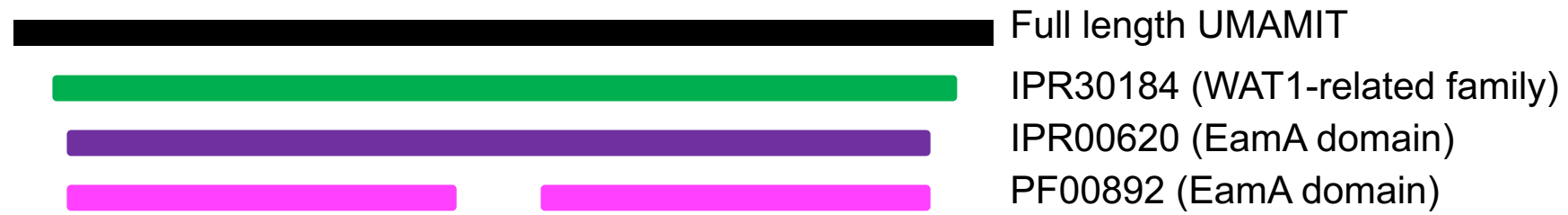

Figure S1. The domain structure of a full-length UMAMIT protein.

Supplement: Supplementary file 1 — Supplementary Figure S1. [file 41598_2021_4284_MOESM1_ESM.pdf]
